# Prospectively Randomized Controlled Trial on Damage Control Surgery for Perforated Diverticulitis with Generalized Peritonitis

**DOI:** 10.1007/s00268-020-05762-1

**Published:** 2020-09-08

**Authors:** Reinhold Kafka-Ritsch, Matthias Zitt, Alexander Perathoner, Elisabeth Gasser, Claudia Kaufman, Sasha Czipin, Felix Aigner, Dietmar Öfner

**Affiliations:** 1 Department of Visceral, Transplant and Thoracic Surgery, Centre for Operative Medicine, Medical University of Innsbruck, Anichstrasse 35, 6020 Innsbruck, Austria; 2 Department of Surgery, Dornbirn General Hospital, Dornbirn, Austria; 3 Department of Surgery, Campus Charité Mitte and Campus Virchow-Klinikum, Charité-Universitätsmedizin Berlin, Berlin, Germany

## Abstract

**Introduction:**

Damage control surgery (DCS) with abdominal negative pressure therapy and delayed anastomosis creation in patients with perforated diverticulitis and generalized peritonitis was established at our Institution in 2006 and has been published. The concept was adopted in other hospitals and published as a case series. This is the first prospectively controlled randomized study comparing DCS and conventional treatment (Group C) in this setting.

**Methods:**

All consecutive patients from 2013 to 2018 with indication for surgery were screened and randomized to Group DCS or Group C. The primary outcome was the rate of reconstructed bowel at discharge and at 6 month. Informed consent was obtained. The trial was approved by the local ethics committee and registered at CinicalTrials.gov: NCT04034407.

**Results:**

A total of 56 patients were screened; 41 patients gave informed consent to participate and ultimately 21 patients (9 female) with intraoperatively confirmed Hinchey III (*n* = 14, 67%) or IV (*n* = 7, 33%), and a median (range) age of 66 (42–92), Mannheim Peritonitis Index of 25 (12–37) and Charlson Comorbidity Index of 3 (0–10) were intraoperatively randomized and treated as Group DCS (*n* = 13) or Group C (*n* = 8). Per protocol analysis: A primary anastomosis without ileostomy (PA) was performed in 92% (11/12) patients in Group DCS at the second-look operation, one patient died before second look, and one underwent a Hartmann procedure (HP). In Group C 63% (5/8) patients received a PA and 38% (3/8) patients a HP. Two patients in Group C, but none in Group DCS experienced anastomotic leakage (AI). ICU and hospital stay was median (range) 2 (1–10) and 17.5 (12–43) in DCS and 2 (1–62) and 22 (13–65) days in group C. In Group DCS 8% (1/12) patients was discharged with a stoma versus 57% (4/7) in Group C (*p* = 0.038, n.s., *α* = 0.025); one patient died before discharge. The odds ratio (95% confidence interval) for discharge with a stoma is 0.068 (0.005–0.861). Intent to treat analysis: A PA was performed in 90% (9/10) of patients randomized to DCS, one patient died before the second look, and one patient received a HP. In group C, 70% (7/10) were treated with PA and 30% (3/10) with HP. 29% (2/7) experienced AI treated with protective ileostomy. In group DCS, 9% (1/11) were discharged with a stoma versus 40% (4/10) in group C (*p* = 0.14, n.s.). The odds ratio for discharge with a stoma is 0.139 (0.012–1.608).

**Conclusion:**

This is the first prospectively randomized controlled study showing that damage control surgery in perforated diverticulitis Hinchey III and IV enhances reconstruction of bowel continuity and can reduce the stoma rate at discharge.

## Introduction

Perforated diverticulitis with generalized peritonitis is still a life-threatening condition requiring urgent surgical intervention. Despite several published randomized trials, showing that primary anastomosis mostly with protective ileostomy is feasible and randomized trials for laparoscopic lavage with conflicting results, the Hartmann procedure is still performed in many hospitals worldwide, leaving a great percentage of patients with a temporary and up to 50% with a permanent stoma. Moreover even protective ileostomies require a second operation and not all of them are taken down [1–9]. Most of these procedures are performed off normal working hours when colorectal and minimal invasive expertise is lacking for decision making regarding anastomosis. Moreover, the course of the septic patient is very hard to estimate intraoperatively. Consequently, the indication to reconstruct bowel continuity in the emergency situation is challenging [10, 11]. On the other hand, the patient needs rapid source control and a Hartmann operation can be a challenging, with complications and sometimes a “bad” stoma, hard-to-tackle.

We established a surgical damage control concept in October 2006 at our academic institution with limited segmental resection or even closure of the perforation site, bridging the patient with open abdomen and negative pressure until a planned second look could be performed under optimized elective conditions and the auspices of a colorectal surgeon. The aim was rapid source control, stabilization of the patient and reconstruction of bowel continuity in the planned second-look operation [12]. This strategy became popular in regional hospitals [13, 14], where a colorectal specialist was less often available, and afterwards in other European hospitals as published in retrospective case series recently [15–19].

To demonstrate the benefit of this concept, e.g. a lower stoma rate, we launched a prospectively randomized controlled study in October 2013 that was planned for a period of five years. The results of this study are presented here.

## Methods

### Study protocol

#### Inclusion criteria

All patients admitted to our department with clinical and radiological suspicion of colonic perforation and generalized peritonitis and who were scheduled for emergency surgery were included in the study.

#### Exclusion criteria

No patient consent.

Age < 18 years.

Pregnancy.

Preoperative anal incontinence.

#### Intraoperative

Covered perforation or peritonitis limited to one quadrant

No colonic perforation (gastric perforation, appendicitis,…)

Malignancy as cause of perforation

#### Primary endpoint

Reconstructed bowel continuity at discharge and at 6 months.

#### Secondary endpoint

Permanent stoma rate.

30-day mortality rate.

Postoperative complications.

All patients preoperatively granted their consent to participate in the study when the indication for emergency surgery was set. After the performing surgeon confirmed colonic perforation with generalized peritonitis, randomization was performed intraoperatively using numbered sealed envelopes prepared by our study centre with computer generated randomization. The envelopes were blocked for ten patients to ensure equal groups.

#### Surgical strategy

In the damage control surgery (DCS) group, the surgeon was asked to perform rapid source control by stapling the perforated segment thus leaving blind ends or by suturing the perforation site if possible, doing a thorough lavage of the abdominal cavity and placing an intra-abdominal negative-pressure system (ABThera KCI^®^, San Antonio, Texas) with 125 mm HG continuously and avoiding retraction of the abdominal wall with dynamic sutures, as published [20]. The second-look operation was scheduled for a time 24–48 h after primary surgery that would be during regular working hours with a colorectal surgeon on hand to make the decision for either anastomosis or ostomy.

In the conventional treatment group (Group C), the perforated colon was resected and bowel continuity reconstructed or a Hartmann procedure was performed immediately. The decision to reconstruct the colon or perform a Hartmann procedure was made by the surgeon on duty during the emergency operation. After performing the anastomosis or the Hartmann procedure, patients with advanced peritonitis received an intra-abdominal negative-pressure system at the discretion of the operating surgeon. This possibility was requested by an intensive care anaesthetist as a member of the local Ethics Committee.

#### Data collection and statistics

Data were collected by our study nurse, who visited the patients, and statistical calculations were performed with SPSS 20. Assuming a reconstruction rate of 80% as published in a prospective cohort study [12, 13] in the study group and 50% in the conventional treatment group, with 20% error of the first kind and a power of 80% testing for superiority of the DCS strategy we calculated (Chi-square analysis) that overall 70 patients would be needed to prove our hypothesis of a lower stoma rate at discharge and at the 6-month control in the DCS group. Statistical calculation was performed with Chi-square or Fisher’s exact test when appropriate for distribution of clinical data and stoma rate, and the Mann–Whitney *U* test was used to compare numeric and nonparametric data. Odds ratio with 95% confidence interval was calculated for the risk of discharge with a stoma. We corrected for multiple testing applying the Bonferroni method, therefore *p*-values < 0.025 rather than 0.05 indicate statistical significance. The patient flow chart is reported as proposed in the CONSORT statement [21].

The study was approved by our local Ethics Committee (EC No.: UN5157) in compliance with the Helsinki Declaration and was planned for a five-year period, registered at CinicalTrials.gov: NCT04034407.

## Results

**Fig. 1:**
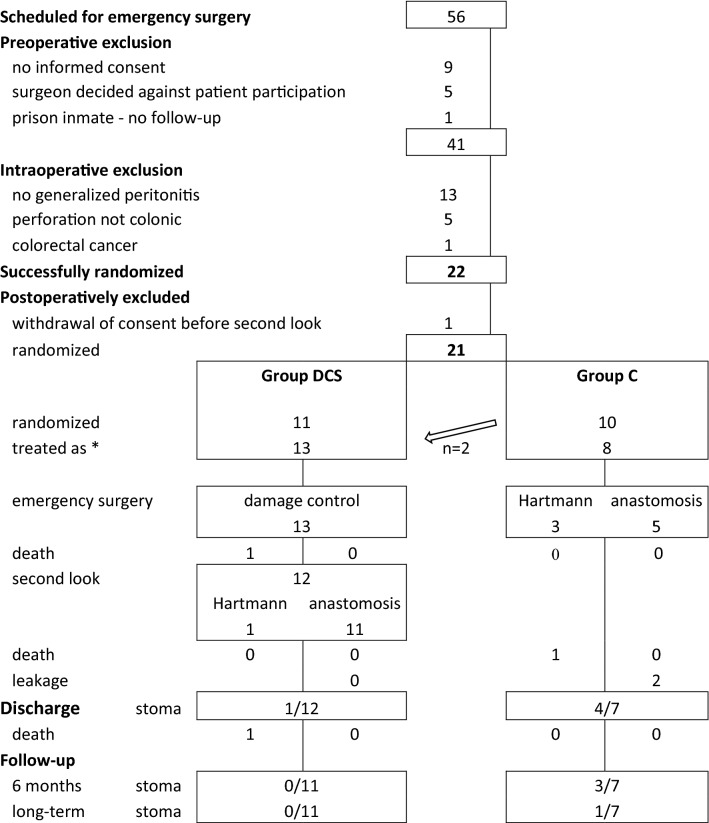
Patient flow chart

Between October 2013 and October 2018, 56 patients were admitted to our hospital and scheduled for emergency surgery for suspicion of perforated diverticulitis with generalized peritonitis established by CT scan and documented in a screening list by the study nurse. In 41 patients, informed consent was obtained. Intraoperatively, 19 patients were excluded (13 for peritonitis involving less than two quadrants, five for perforation not of colonic origin, one patient because of malignancy). Twenty-two patients were randomized, but one patient withdrew consent before the second-look operation. Two patients randomized to Group C were treated with the damage control concept for ethical reasons. In view of the deterioration after laparotomy both the surgeon and the anaesthetist felt it was necessary to perform a damage control operation (see Fig. 1: Flow chart).

## Per protocol PP analysis

At the planned second-look operation under elective conditions, bowel reconstruction was realized without a protective ileostomy in 11 (92%) of the 12 patients treated with DCS. Four of these (34%) patients had faecal peritonitis and in one patient (92%) a Hartmann procedure was performed. The decision to reconstruct the colon or perform a Hartmann procedure was made by a colorectal surgeon, who at least supervised the procedure. No anastomotic leakage was observed in this group.

In five of the eight patients (34%) in Group C, the control group, an anastomosis was performed during initial surgery. The decision to reconstruct the colon or perform a Hartmann procedure was made by the general surgeon on duty. Two of these patients (40%) experienced minor leakage, which was managed with a re-laparotomy, repair of the anastomosis and a loop ileostomy. One of the patients with a Hartmann procedure died before discharge, and four of the remaining seven patients (57%) were discharged with a stoma (2 Hartmann, 2 ileostomies) versus one (8%) of the 12 surviving patients in Group DCS (*p* = 0.038; Bonferroni correction for multiple testing requires a *p* value < 0,025). The calculated odds ratio (95% confidence interval) for discharge with a stoma is 0.068 (0.005–0.861).

During follow-up, the patient discharged with a colostomy in Group DCS died because of a progressive lymphoma, leaving 11 (100%) patients without a stoma at the 6-month control. In Group C one patient underwent reversal of the ileostomy before six months, leaving three of the seven (43%) Group C patients with a stoma (*p* = 0.036). In two patients the stoma was reversed later than six months, leaving one patient with a permanent stoma.

No statistical difference was found in the outcome between the two groups in terms of mortality, ICU or hospital stay (see Tables 1 and 2). A trend towards a higher rate of postoperative organ dysfunction was observed with 4/8 in Group C versus 2/13 in Group DCS (*p* = 0.08) (see Table 2).

**Table 1:** Demographic data: per protocol analysis

		Study group	Control
Age	Median (range)	66 (45–92)	67 (42–83)
Female	*n*/*n* (%)	6/11 (46%)	3/8 (38%)
Charlson Comorbidity Index	Median (range)	2 (0–10)	3.5 (1–8)
Hinchey IV (faecal)	*n*/*n* (%)	4/11 (31%)	3/8 (38%)
Mannheim peritonitis index	Median (range)	26 (12–37)	24.5 (16–28)
Laboratory on admission	Mean (SD)		
CRP on admission		16 (± 12)	11 (± 11)
Leukocytes G/l		12.4 (± 2.4)	12.5 (± 3.2)
Haemoglobin g/l		13.6 (± 2.4)	12.7 (± 3.2)
Creatinine mg/dl		1.3 (± 0.64)	1.8 (± 2.3)
Bilirubin mg/dl		1.2 (± 1.0)	1.5 (± 1.7)
PTT		39 (± 16)	40 (± 14)
Protein g/dl		6.3 (± 1.0)	7.2 (± 1.4)
Lactate mg/dl		12.5 (± 4.3)	12.5 (± 8.5)

*n*, number, *SD* standard deviation, *CRP* C-reactive protein, *PTT* partial thromboplastin time

**Table 2:** Outcome: per protocol analysis

		Study group	Control
Anastomosis	*n*/*n* (%)	11/12 (92%)	5/8 (63%)
Anastomotic leakage		0/11 (0%)	2/5(40%)
Discharge with stoma		1/12 (8.3%)	4/7 (57%)
Postoperative organ dysfunction		2/13 (15%)	4/8 (50%)
Complication Clavien–Dindo > = 3		4/13 (31%)	4/8 (50%)
Cardiac complication		1/13 (8%)	1/8 (13%)
SSI		6/12 (50%)	4/8 (50%)
ICU stay	Median (range)	2 (1–20)	2 (1–62)
Hospital stay		17,5 (12–43)	22 (13–65)
30-day mortality		1/13 (8%)	1/8 (13%)
Stoma at 6 months		0/11 (0%)	3/7 (27%)
CRP day 3	Mean (SD)	18 (± 6)	18 (± 11)
CRP day 5		17 (± 8)	12 (± 9)

*n* number, *SSI* surgical site infection, *ICU* intermediate care unit, *CRP* C-reactive protein

### Intent-to-treat analysis

Of the 11 patients randomized to DCS surgery, one patient died before the second-look operation, in nine patients (90%) bowel reconstruction was realized without a protective ileostomy, and in one patient (10%) a Hartmann procedure was realized. No anastomotic leakage was observed in this group. During follow-up the patient discharged with a colostomy in Group DCS died because of a progressive lymphoma, leaving nine patients alive and all without a stoma at the 6-month follow-up.

In three of the ten patients in Group C (30%), the control group, a Hartmann procedure was performed during initial surgery, in five patients an anastomosis was created during initial surgery and in two patients at the second-look operation, as they had been treated with damage control, as mentioned above. Two of the patients experienced minor leakage (20%), which was managed with re-laparotomy, repair of the anastomosis and loop ileostomy. One patient died for severe sepsis during hospital stay. Thus, four of the nine patients (44%) were discharged with a stoma versus one (10%) in the DCS group (*p* = 0.14). The calculated odds ratio for discharge with a stoma is 0.139 (0.012–1.608). In one of them ileostomy closure was performed before the 6-month follow-up, in two of them the stoma was closed later, leaving three patients with a stoma at the 6-month follow-up (*p* = 0.08) and one patient with a permanent stoma (see Table 3).

**Table 3:** Outcome: intent to treat analysis

		Study group	Control
Anastomosis	*n*/*n* (%)	9/11 (90%)	7/10 (70%)
Anastomotic leakage		0/9 (0%)	2/7(29%)
Discharge with stoma		1/11 (10%)	4/10 (40%)
Postoperative organ dysfunction		2/11 (19%)	4/10 (40%)
Complication Clavien–Dindo > = 3		4/11 (36%)	4/10(40%)
Cardiac complication		1/11 (9%)	1/10 (10%)
SSI		4/11 (36%)	6/10 (60%)
ICU stay	Median (range)	2 (1–20)	4 (1–62)
Hospital stay		18,5 (12–43)	18 (13–65)
30-day mortality		1/11 (9%)	1/10 (10%)
Stoma at 6 months		0/10 (0%)	3/9 (27%)
CRP day 3	Mean (SD)	18 (± 6)	17 (± 11)
CRP day 5		16 (± 3,4)	15 (± 11)

*n* number, *SSI* surgical site infection, *ICU* intermediate care unit, *CRP* C-reactive protein

Negative abdominal pressure therapy was prolonged for two more days in one patient in the DCS group. This was the patient with the Hartmann procedure, where advanced peritonitis was observed. In the control group five patients received an abdominal negative-pressure system for two days to treat intra-abdominal sepsis. In all patients, direct abdominal wall closure was performed. No side effects of open abdomen treatment such as enteroatmospheric fistula were observed.

After the end of the planned and approved five years, the study period was not extended for various reasons. To reach the initially calculated number of patients up to ten years more would have been necessary. Moreover, evidence and expertise for minimal invasive surgery for perforated diverticulitis has changed over time and we decided to integrate minimal invasive surgery in our damage control concept in a planned multicentre study.

## Discussion

Damage control surgery for patients with a life-threatening emergency like perforated diverticulitis with generalized peritonitis implies several advantages [10]. Beside rapid source control, avoiding an aggravation of abdominal sepsis by mobilizing the colon and allow resuscitation of the patient, the decision to reconstruct colon continuity or not can be postponed and made after stabilizing the patient under elective conditions under the auspices of a colorectal surgeon. Especially in smaller hospitals with reduced staff or working hours, this concept was rapidly adopted. The feasibility of our concept has been demonstrated in other institutions, as recently published in retrospective series [15–19]. Even the Hartmann procedure itself can be challenging and stoma complications or “bad” stomas requiring surgical revision are frequent [22, 23]. Ostomies have great impact on quality of life as well as nursing-related costs and reversal is associated with significant morbidity [24–27].

To our knowledge, this is the first description of randomized controlled data regarding the damage control concept. Despite the difficulty of recruiting a sufficient number of patients, we demonstrate a lower stoma rate at discharge in the per protocol treated patients in Group DCS. No statistical difference in mortality or morbidity was observed between the study groups. Nevertheless, this study is underpowered for the purpose of demonstrating an effect of the concept on postoperative recovery. At least a trend towards fewer patients with postoperative organ dysfunction was observed.

One reason for the small number of included patients is the fact that the indication for surgery was strictly set in this clinical setting, as also described in recent publications [26, 28]. Clinically stable patients with limited free air were treated with antibiotics and patients without generalized peritonitis were intraoperatively excluded [29]. Otherwise, in unstable patients with acute abdomen it is not always possible to obtain informed consent, especially when patients are on opioids for pain control. Tartaglia et al. reporting on a multicenter case series ranging over a period of six years were able to include 34 patients in a similar setting. Our study was terminated after the initially approved period of five years and not prolonged because it would have taken at least ten years more to reach the calculated number of overall 70 patients. Moreover, as evidence for laparoscopic surgery and minimal invasive methods in perforated diverticulitis is changing, we will integrate minimal invasive strategies for selected patients when colorectal expertise is available in our future treatment algorithm. Nevertheless, we were able to confirm a higher rate of bowel reconstruction with the concept of initial damage control surgery, and to our knowledge this is the first such randomized study.

Another shortcoming of our study is the fact that for medical reasons two patients were not treated with definitive surgery in the initial operation despite randomization to Group C. The experience of eight years of successful damage control surgery before establishing this randomized controlled study at our department led the surgeon and anaesthetist in charge to violate the protocol for ethical reasons, thus avoiding harm to the patient from a prolonged operation.

Based on his daily experience at the intensive care unit, the anaesthetist on our local Ethics Committee requested the possibility for negative abdominal pressure therapy, even for the control group, for the case of severe abdominal sepsis or advanced peritonitis. This therapy was applied in five out of ten patients in the control group. This reduces our chance to demonstrate an effect of the intra-abdominal negative-pressure system on postoperative recovery. Nevertheless, the study still demonstrates that the decision to reconstruct the colon is more frequently made in the second-look operation under elective conditions with a colorectal surgeon available. So considering a damage control surgery instead of performing a Hartmann´s procedure is a probable option.

It is important to note that patients treated with the surgical damage control concept with open abdomen and negative pressure can be extubated and mobilized as published before.[12, 13]. Delayed surgery might constitute overtreatment for some patients, but sepsis development is unpredictable and a Hartmann procedure always requires a second operation anyway [30].

Laparoscopic lavage was proposed for patients in this setting despite the fact that conflicting results have been reported [23, 31–35]. We have little experience, because patients with only some free air bubbles or stable patients are treated conservatively or with percutaneous drainage at our department, as proposed in recent publications [28]. Moreover, in this situation laparoscopy demands experience and is therefore not applicable for emergencies at night or on weekends, whereas a simple laparotomy, lavage and limited closure of a colon perforation can be performed even by general surgeons with little or no colorectal experience. Faecal peritonitis turned out to be a contraindication for laparoscopic lavage [31]. In our study, faecal peritonitis was not a contraindication for anastomosis and did not result in a higher leakage or stoma rate.

In conclusion, we demonstrate control surgery and delayed creation of bowel continuation under elective conditions can be an option to avoid a stoma and should be taken into consideration before realizing a Hartmann´s procedure. This might play an important role in times of working hour restrictions and reduced availability of specialists, but a multicentre study with more power should confirm these first and limited randomized data.

## Abbreviations

CRP: C-reactive protein
DCS: Damage control surgery
HP: Hartmann’s procedure
ICU: Intensive care unit
MPI: Mannheim Peritonitis Index
n: Number
NPT: Negative-pressure therapy
PA: Primary anastomosis without ileostomy
PTT: Partial thromboplastin time
SD: Standard deviation
SSI: Surgical site infection
